# Phytochemical Study of Aqueous Extract of* Ochna schweinfurthiana* F. Hoffm Powder Bark and Evaluation of Their Anti-Inflammatory, Cytotoxic, and Genotoxic Properties

**DOI:** 10.1155/2019/8908343

**Published:** 2019-02-18

**Authors:** Steve V. Djova, Maximilienne A. Nyegue, Angelique N. Messi, Alian D. Afagnigni, François-X. Etoa

**Affiliations:** ^1^Department of Biochemistry, University of Yaounde I, PO Box 812, Yaounde, Cameroon; ^2^Department of Microbiology, University of Yaounde I, PO Box 812, Yaounde, Cameroon; ^3^Department of Organic Chemistry, University of Yaounde I, PO Box 812, Yaounde, Cameroon

## Abstract

*Ochna schweinfurthiana *has been used in traditional medicine to treat pain, inflammation, and arthritis. It is a rich source of complex dimers of flavonoids with potential use as templates for the development of therapeutic drugs. Hence, the aim of this study was to study the phytochemical content and evaluate the in vitro cytotoxic, genotoxic, and anti-inflammatory activities of the aqueous extract of* Ochna schweinfurthiana *bark (OSE). Phytochemical study was carried out according to LC-MS procedures, while isolation was carried out using thin layer and column chromatographies. Cytotoxicity was investigated by the mitochondrial viability [3-(4,5-dimethylthiazol-2-yl)-2,5-diphenyltetrazolium bromide] (MTT) method while genotoxicity potential of the extract was ascertained using the* Salmonella typhimurium *test strains TA98 and TA100. The anti-inflammatory effect of OSE was evaluated by the in vitro inhibition of 15-lipooxygenase enzyme and bovine serum albumin denaturation (BSA) assays. The investigation of compounds extracted from OSE led to the identification and isolation of six known compounds, namely, hemerocallone (9), 6,7-dimethoxy-3'-4'-dimethoxyisoflavone (10), lithospermoside (13), amentoflavone (14), agathisflavone (15), and *β*-D-fructofuranosyl-*α*-D-glucopyranoside (17). In the anti-inflammatory assay, aqueous extracts of the bark showed selective inhibition of 15-lipooxygenase with IC_50_ value of 32.2 ± 0.36  μg/mL and the result of the bovine serum albumin denaturation assay with IC_50_ value of 130± 5.78 *μ*g/mL showed moderate activity. The toxicity assay indicated that OSE are noncytotoxic on Vero cell line with LC_50_ value of 50 mg/mL and nongenotoxic toward* Salmonella typhimurium *tester strain TA98 and TA100. Result from this study supports the traditional use of the selected medicinal plants in Cameroon for the treatment of inflammatory conditions. Noncytotoxicity and nongenotoxicity of OSE suggest that this plant is safe for use.

## 1. Introduction

Inflammation is a complex reaction of vascularised tissues to infection, toxin exposure, or cell injury which involves extravascular accumulation of immune cells. The mechanisms of inflammation are of great benefit in the maintenance of body homeostasis. The inflammatory response may be physiologically appropriate in the presence of an infection and cellular damage or stress. Conversely, it may be inappropriate, pathologic, and damaging altering homeostasis, when it is reacting out of proportion thus contributing to diseases [1, 2]. In effect, inflammation is an important baseline reaction responsible for manifestations of various chronic diseases such as cancer, septic shock, diabetes, atherosclerosis, obesity, cardiovascular disease, age-related molecular degeneration, chronic obstructive pulmonary disease, and multiple sclerosis [3]. The mechanisms of inflammation involve a series of events in which the metabolism of arachidonic acid plays an important role. It is metabolised through several pathways. In path 1: the addition of 2 moles of oxygen to arachidonic acid, catalysed by cyclooxygenases (COX-1 and COX-2), leads to the formation of a tricyclic endoperoxide whose reduction gives a prostaglandin endoperoxide H_2_PGH_2_. PGH_2_ is an unstable intermediate from which various prostanoid structures are formed according to several enzymatic reactions: prostaglandins, prostacyclins, and thromboxanes A2 [4]. In the second pathway, it is initiated by the action of lipoxygenases on arachidonic acid producing trioxilins and leukotrienes. Several enzymes act in this way: 5-lipoxygenase which reduces arachidonic acid to 5-hydroperoxy-eicosatetraenoic (5-HPETE) from which leukotrienes (LT) are obtained; 12-lipoxygenase which will reduce arachidonic acid to 12-hydroperoxy-eicosatetraenoic (12-HPETE) which will subsequently give the hepoxilins A3/B3 from which hepoxilin hydrolases will release the trioxilin A3/B3; and finally 15-lipoxygenase which will reduce arachidonic acid to 15-hydroperoxy-eicosatetraenoic (15-HPETE) from which lipoxins A/B are released. Prostaglandins, prostacyclins, thromboxanes A2, leucotrienes, and trioxilin A3/B3 are important biologically active mediators in a variety of inflammatory events [5]. Inflammation is also induced during the increase in vascular permeability and increase in protein denaturation and membrane alteration. Protein denaturation is a process in which proteins lose their tertiary structure and secondary structure by application of external stress or compounds, such as strong acid or base, a concentrated inorganic salt, an organic solvent or heat. Most biological proteins lose their biological function when denatured. Denaturation of protein is a well-documented cause of inflammation [6, 7].

Fortunately, the treatment of arthritis and other inflammatory disorders involves the use of different classes of drugs such as nonsteroidal anti-inflammatory drugs (NSAIDs), corticosteroids, and disease modifying antirheumatic drugs (DMARDs). But the use of NSAIDs has gastrointestinal side effects, which includes irritation of the gastric mucosa, belching, gastric ulceration, and bleeding. Long-term use of NSAIDs may impair renal and hepatic functions, predisposing the patient to cardiovascular diseases [8]. In the same way, herbal medicines can be potentially toxic to human health, based on their long-term use. Many plants used in traditional and folk medicine are potentially cytotoxic, genotoxic, mutagenic, and carcinogenic [9, 10]. Assessment of the potential cytotoxicity and genotoxicity of traditional medicines is indeed an important issue as damage to the genetic material may lead to critical mutations and to an increased risk of cancer and other diseases. Hence, it is urgent to explore alternative drugs from plants and their toxicological investigations.

*Ochna schweinfurthiana *F. Hoffm is a tropical small tree that measures upto 4 metres and belongs to the Ochnaceae family. It is used by the population of North Cameroon to treat different metabolic diseases involving mechanisms of oxidation or inflammation such as rubella, burns, stomach ache, and multiple sclerosis [11]. Several studies revealed that the Ochnaceae family is a rich source of complex metabolites such as flavonoids, chalcones, steroids, terpenoids, and alkaloids [12–14]. The antimicrobial effect of methanol and acetone extracts of the leaves of* O. schweinfurthiana* has been reported [15]. The evaluation of the antioxidant activity of the leaves, stem-barks extracts, and fractions of* O. schweinfurthiana* has been already done [16]. An earlier study reported on the phytochemistry of the stem bark of* O. schweinfurthiana* and demonstrated cytotoxicity against HeLa cells of the methanolic and ethyl acetate extracts, as well as the isolation of amentoflavone and agathisflavone [17]. Messi et al. [18] isolated three new antiplasmodial and antioxidant agents from the roots of* O. schweinfurthiana*. To date and to the best of our knowledge, no data are available on the anti-inflammatory and toxicological potential of* O. schweinfurthiana*. Therefore, the aim of this study was to investigate the phytochemical content of the aqueous extract of* Ochna schweinfurthiana *bark powder and evaluate their anti-inflammatory, cytotoxic, and genotoxic properties.

## 2. Materials and Methods

### 2.1. Plant Material and Extraction Procedure

The botanical material commonly known in Cameroon as* Sa'aboule* in fulfulde is constituted of barks. It was harvested in August 2014 in Ngaoundere (Adamawa, Cameroon) and identified at the National Herbarium of the Institute of Agricultural Research for Development (IRAD, Yaounde, Cameroon) under the identification code: 40171HNC.

**Figure 1:**
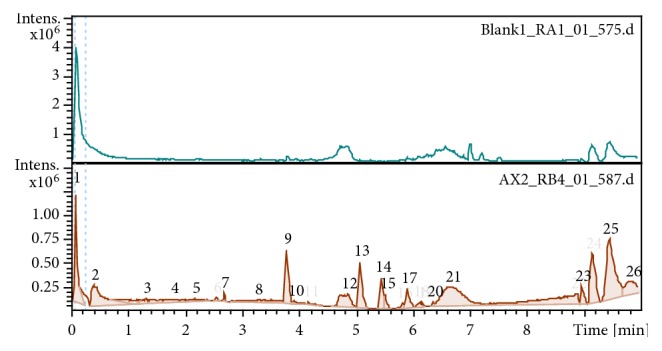
LC-MS chromatogram obtained from OSE.

The barks of* O. schweinfurthiana* were cut into small sizes with a knife and air-dried on clean tarpaulins for one week at room temperature and weighed. The sufficiently dried bark was coarsely ground in an electrical blender. The resulting powder (400 g) was extracted 3 times with 5 L of water using the Biobase lyophilizer to yield a crude extract of (120 g). The resulting extract was analyzed using HPLC-MS as shown in Figure 1 and partitioned with n-hexane and ethyl acetate to yield two portions.

The hexane portion (300 mg) mostly containing fats was not used in this study. However, the ethyl acetate portion (1033 mg) showed the presence of compounds 9, 10, 13, 14, 15, and 17, which were earlier identified in the* O. schweinfurthiana* stem bark of ethyl acetate extract [17]. This portion was subjected to chromatography over silica gel, eluting with gradients of CH_2_Cl_2_/MeOH to produce 75 fractions combined on the basis of their TLC profiles into 3 fractions: A (96 mg; 1-25), B (160 mg; 26-50), and C (200 mg; 51-75).

Fraction A (CH_2_Cl_2_/MeOH; 30/1) was purified by silica gel column chromatography with gradients (CH_2_Cl_2_/MeOH; 30/1 and 20/1) to yield compound 17 (25 mg).

Fraction B (CH_2_Cl_2_/MeOH; 20/1) was purified by silica gel column chromatography with gradients (CH_2_Cl_2_/MeOH; 20/1; 10/1 and 8/1) to yield compounds 13 (7.24 mg), 14 (61 mg), and 15 (2.5 mg).

Fraction C (CH_2_Cl_2_/MeOH; 5/1) was purified by column chromatography on sephadex LH-20 with gradient (CH_2_Cl_2_/MeOH; 5/1) to give compounds 9 (75 mg) and 10 (13.6 mg).

### 2.2. LC-MS Procedures

LS-MS analysis of OSE was carried out following a modified method of Abay et al. [19] as described previously by Gheorghe et al. [20]. C_18_ reversed-phase column oven (30°C) was used in this study.

### 2.3. Anti-Inflammatory Assay

#### 2.3.1. Ferrous Oxidation-Xylenol Orange (Fox) Assay

The assay was performed according to Pinto et al. [21] and Delong et al. [22] with slight modifications as described previously by Dzoyem and Eloff [23].

#### 2.3.2. Bovine Serum Albumin (BSA) Denaturation Assay

Protein denaturation was performed as described by Sakat et al. [24] with slight modifications. The test solution consisting of 1 mL of different concentrations of extracts preparation ranging from 1000-50 *μ*g/mL or standard sodium diclofenac 100 and 250 *μ*g/mL was mixed with 1 mL of egg albumin solution (1 mM) and incubated at 27 ± 1°C for 15 min. Denaturation was induced by keeping the reaction mixture at 70°C in a water bath for 10 min. After cooling, the turbidity was measured using the Jenway 6305 spectrophotometer at 660 nm. Percentage inhibition of denaturation was calculated from control where no drug was added. Each experiment was done in triplicate.

### 2.4. Genotoxicity Test

The potential genotoxic effects of the barks from* O. schweinfurthiana* were investigated using the* Salmonella typhimurium *test strains TA98 and TA100 according to Maron and Ames [25] as described previously by Makhafola et al. [26].

### 2.5. In Vitro Cytotoxicity Testing of Biologically Active Extracts Using the MTT Assay

The MTT assay according to Mosmann, [27] and McGaw et al. [28] with some minor modifications as described previously by Madizela et al. [29] was used to evaluate viability of cells after their exposure to the test substances. The Vero (African green monkey kidney) cell line obtained from the Department of Veterinary Tropical Diseases, University of Pretoria, South Africa, was used to investigate the cytotoxicity of the biologically active extracts.

### 2.6. Statistical Analysis

The results are presented as means of three experiments. Statistical significance between groups was calculated by using a paired t-test with GraphPad Prism software (version 7). Values were expressed as mean ± SD and differences were considered significant statistically if* P*<0.05.

## 3. Results

### 3.1. Phytochemicals Screening

The compounds constituting the OSE were identified through interpretation of their mass spectrum obtained by LC/MC in comparison with previously reported data from the literature. The compounds were identified from their protonated molecular ion [M]^ +^. Figure 1 resumes the number of phytoconstituents present in the extract by comparing with the blank.

**Scheme 1:**
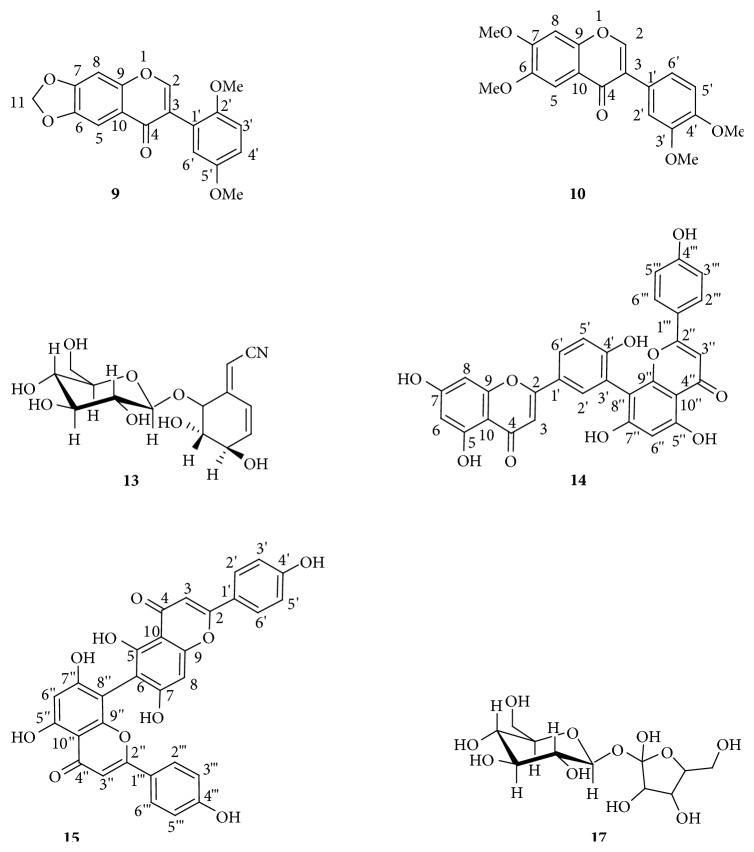
The chemical structures of 6 known compounds isolated from bark aqueous extract of Ochna schweinfurthiana.

OSE exhibited the presence of six compounds (9, 10, 13, 14, 15, and 17), which were also isolated and identified in the* O. schweinfurthiana* stem bark of ethyl acetate extract [17].* Six Known Compounds.* (1) Hemerocallone** (9)**, it exhibited a pseudomolecular ion peak at m/z 326.1428 [M]^+^ in the HRESIMS corresponding to the molecular formula C_18_H_14_O_6_ and retention time of 3.8 min; all these physical and spectroscopic data are very similar to those of previously reported data [17, 30]. (2) 6,7-Dimethoxy-3'-4'-dimethoxyisoflavone** (10)**, the positive ion mass spectrum exhibited a pseudomolecular ion peak at m/z 342.1021 [M]^+^ in the HRESIMS suggesting the molecular formula C_19_H_18_O_6_ and retention time of 3.9 min; this compound was previously isolated and identified by Ortega et al. [31] and Ndongo et al. [17]. (3) lithospermoside** (13)** showed a pseudomolecular ion peak at m/z 329.1248 [M]^+^ in the HRESIMS, consistent with the molecular formula C_14_H_19_NO_8_ and retention time of 5.2 min, also identified by Quanbin et al. [32] and Ndongo et al. [17]. (4) Amentoflavone** (14)** showed a pseudomolecular ion peak at m/z 537.0826 [M+H]^+^ in the HRESIMS corresponding to the molecular formula C_30_H_18_O_10_, with retention time of 5.2 min; all these physical and spectroscopic data are very similar to those of previously reported data [17, 33]. (5) Agathisflavone** (15)** exhibited a pseudomolecular ion peak at m/z 539.2010 [M+H]^+^ in the HRESIMS corresponding to the molecular formula C_31_H_30_O_10_ and retention time of 5.3 min which was isolated and identified by Souza et al. [34] and Ndongo et al. [17]. (6) *β*-D-Fructofuranosyl-*α*-D-glucopyranoside** (17)** showed a pseudomolecular ion peak at m/z 666.2125 [M+H]^+^ in the HRESIMS corresponding to the formula C_24_H_42_O_21_ and retention time of 5.8 min; this compound was isolated and identified by Tasuya et al. [35] and Ndongo et al. [17]. The structures of the isolated compounds were elucidated using MS and NMR spectroscopy by comparisons with previously reported data (Scheme 1).

### 3.2. MTT Assay

A lot of attention is devoted to cytotoxicity studies as a first research step in toxicity evaluation of plant extract and active compounds isolated from plants. Cytotoxicity was determined using MTT [3-(4,5-dimethylthiazol-2-yl)-2,5-diphenyltetrazolium bromide] assay on Vero monkey kidney cell line. According to the US NCI plant screening program, a crude extract is generally considered to have* in vitro* cytotoxic activity if the IC_50_ value following incubation between 48 and 72 h is less than 20 *μ*g/mL, while it is less than 4 *μ*g/mL for pure compounds [36]. The results of the cytotoxicity test showed that aqueous extracts of bark incubated after 48 h with Vero monkey kidney cell line had a lethal concentration (LC_50_) of 50±1 *μ*g/mL, so the aqueous extracts of OSE are not cytotoxic on the Vero monkey kidney cell line. However, more toxicity studies are required to evaluate the safety of this plant.

### 3.3. Genotoxicity Assay

According to the literature, in the presence of crude plant extracts, a transformation of* Salmonella typhimurium* strain TA98 from HIS^−^ to HIS^+^ by spontaneous reversion between base pair showed positive results, whereas the substitution of a base pair on the* Salmonella typhimurium* strain TA100 highlighted the positive results of the crude extracts of plants [37]. To be considered genotoxic, the increase of revertants number must be proportional to the dose of the extracts evaluated; in other words, the number of colonies obtained by the genotoxic effect of the extracts evaluated must be equal to or greater than twice the number of colonies obtained by the genotoxic effect of the negative control (Table 1) [25]. It appears from this study that OSE extracts are nongenotoxic because OSE had not demonstrated a dose-dependent increase, or revertant colonies equal to or greater than twice the number of negative control revertant colonies. This result showed that OSE is devoid of any genotoxic substances that can lead to mutations by reversion or substitution in the genetic material of an organism.

### 3.4. Anti-Inflammatory Activity

#### 3.4.1. Ferrous Oxidation-Xylenol Orange (Fox) Assay

**Figure 2:**
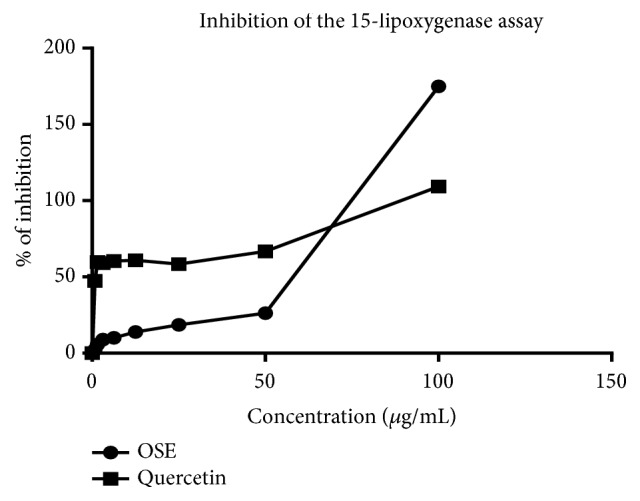
% of inhibition of the 15-lipoxygenase activity of OSE and Quercetin at different concentrations from which IC_50_ values of OSE and Quercetin were obtained. Values are mean ±SD of three experiments.

**Figure 3:**
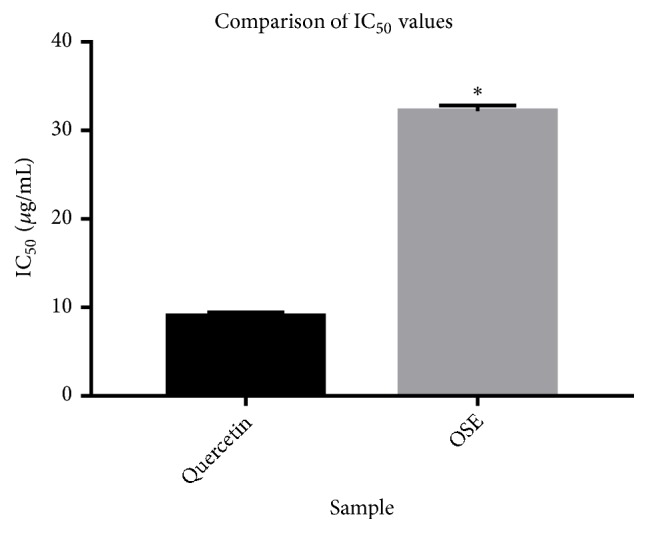
Comparison of IC_50_ of OSE with Quercetin. Data are expressed as mean ± SD; Quercetin was used as a reference compound. Statistical differences between quercetin and OSE as analyzed by the paired t-test (^*∗*^*P* < 0.0001).

The effects of* O. schweinfurthiana *bark extracts on the production of leukotrienes were determined through the inhibition of 15-lipoxygenase activity and the results are shown in Figures 2 and 3. The results suggest that aqueous bark extract has got good 15-lipoxygenase inhibitory activity with an IC_50_ value of 32.2 ± 0.36 *μ*g/mL compared to the standard quercetin with and IC_50_ value of 9.013±0.25 *μ*g/mL (P <0.0001).

#### 3.4.2. BSA Denaturation Assay

**Figure 4:**
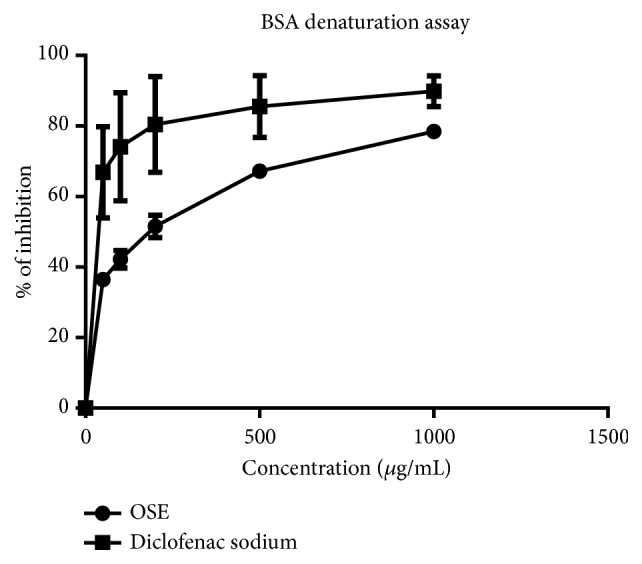
% of inhibition of the protein denaturation activity of OSE and Diclofenac sodium at different concentrations from which IC_50_ value of OSE and Diclofenac sodium was obtained. The error bars represent the standard deviation of measurement of the absorbance; experiment was done in triplicate.

**Figure 5:**
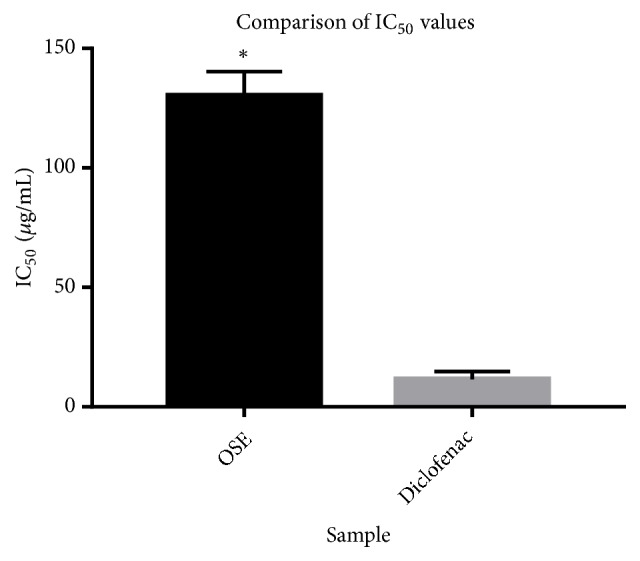
Comparison of IC_50_ between OSE and diclofenac sodium. Data are expressed as mean ± SD, sodium Diclofenac was used as a reference compound. Statistical differences between sodium diclofenac and OSE as analyzed by the Student t-test (^*∗*^*P* < 0.0001).

The aqueous extract of* O. schweinfurthiana *was analyzed for its BSA denaturation activity and is compared with that of sodium diclofenac as the standard. From the results, it can be stated that the aqueous extract of* O. schweinfurthiana* with IC_50_ value of 130±5.78 *μ*g/mL is effective in inhibiting heat induced albumin denaturation compared with that of the standard sodium diclofenac (IC_50_ of 11.53± 1.92 *μ*g/mL) (Figures 4 and 5,* P<0.0001*).

## 4. Discussion

Pharmaceutical analysis is fundamental in the discovery and development of new drugs. Such analysis cannot be performed without the development of LC especially LC-MS. LC-MC usually acts as equipment for identification and as a device for sample clean-up. LC-MS is dominantly preferred for pharmaceutical analysis [38]. We report the identification and isolation of hemerocallone; 6,7-dimethoxy-3'-4'-dimethoxyisoflavone; lithhospermoside; amentoflavone; agathisflavone; and *β*-D-fructofuranosyl-*α*-D-glucopyranoside in OSE. This result confirms that of Pegnyemb et al. [14] and Abdullahi et al. [11] who revealed that Ochnaceae family is rich in complex dimers of flavonoids and chalcones. Previous phytochemical studies of the bark of* Ochna schweinfurthiana* led to the identification and isolation of flavonoids and glucosides from leaves and bioflavonoids from the roots Pegnyemb et al. [14]. Recently, amentoflavone and agathisflavone were isolated from the stem bark of* O. schweinfurthiana* [17, 18]. The anti-inflammatory effect was ascertained in this study by the inhibition of 15-lipoxygenase and bovine serum albumin (BSA) denaturation assays. OSE had good activity against inhibition of the 15-lipoxygenase enzyme. However, the positive control (Quercetin) with IC_50_ value of 9.013 *μ*g/mL was better than the OSE which had IC_50_ value of 32.2 *μ*g/mL. The promising antilipoxygenase activity of OSE may be linked to the presence of flavonoids [39]. Flavonoids are known to interfere with the different stages of the arachidonate cascade via cyclooxygenase or lipoxygenase pathways to alleviate inflammatory responses [40]. This result supports the traditional use of the selected medicinal plants in Cameroon to the management of inflammatory conditions. OSE moderately inhibited bovine albumin denaturation with an IC_50_ value of 130 *μ*g/mL and was less than that of the standard diclofenac sodium which had IC_50_ value of 11.53 *μ*g/mL. Most of the investigations have reported that when BSA is heated, it undergoes denaturation and antigens are expressed which are associated with type-III hypersensitivity reaction, which in turn is related to disease such as rheumatoid arthritis [41]. Mechanism of denaturation probably involves alterations in electrostatic, hydrogen, hydrophobic, and disulphide bonding [40]. From the results of the present study, it can be stated that OSE is capable of controlling the production of autoantigen and inhibits denaturation of protein in rheumatic disease. With regard to* in vitro* cell culture systems, when a substance interferes with the attachment of cells, it alters the morphology and the rate of cell growth or causes them to die, it is then considered to be cytotoxic [42]. According to the US NCI plant screening program, a crude extract is generally considered to have in vitro cytotoxic activity if the IC_50_ value following incubation between 48 and 72 h is less than 20 *μ*g/mL, while it is less than 4 *μ*g/mL for pure compounds [37]. The results of the cytotoxicity test showed that aqueous extracts of bark incubated after 48 h with Vero monkey kidney cell line had a lethal concentration (LC_50_) of 50 *μ*g/mL, so the aqueous extract of OSE is not cytotoxic on the Vero monkey kidney cell line. For more in-depth investigation, subsequent tests should be conducted with other methods such as the lactate dehydrogenase (LDH) leakage, protein quantification, or neutral red. Assessment of the potential genotoxicity of traditional medicines is indeed an important issue as damage to the genetic material may lead to critical mutations and therefore also to an increase risk of cancer and other diseases. This is true also when evaluating the potential of DNA damaging effects of plant extracts containing a plethora of more or less potent bioactive compounds [39]. In our study, the tested extract did not demonstrate a dose-dependent increase or revertant colonies that were equal to or greater in number than twice those of the negative control. Therefore, the tested plant extract lacked direct genotoxic compounds. The possible ways in which inhibitors of genotoxic agents can act include the inhibition of interaction between genes and biochemically reactive genotoxic agent and the inhibition of metabolic activation of indirectly acting toxicants [43].

## 5. Conclusion

The in vitro study of aqueous extract of* O. schweinfurthiana* revealed promising anti-inflammatory activity. The study corroborates traditional claims of the use of these Cameroonian medicinal plants in the management of arthritis, infections, rheumatism, and inflammation. The isolation and identification of compounds such as hemerocallone, 6,7-dimethoxy-3'-4'-dimethoxyisoflavone, lithhospermoside, amentoflavone, agathisflavone, and *β*-D-fructofuranosyl-*α*-D-glucopyranoside in aqueous extract of bark demonstrated that* O. schweinfurthiana* have potential for development as therapeutic agents of inflammation. Noncytotoxicity and nongenotoxicity of OSE suggest that this plant is probably safe for use; also, subsequent tests should be conducted with other methods to confirm the low cytotoxicity of the OSE.

## Figures and Tables

**Table 1:** Summary of genotoxicity assay.

Samples	Concentrations (mg/mL)	*Salmonella typhimurium* strains	
		TA98	TA100
Aqueous extract bark	5	20 ± 0.57	119 ± 2
*A. schweinfurthii*	0.5	18.66 ± 0.33	126.00 ± 2
A	0.005	9.33 ± 0.33	131 ± 2.33
4NQO		133 ± 0.57	95.66 ± 0.66
Water		22.00 ± 0.57	104.00 ± 2.00

4NQO: positive control, water: negative control.

## Data Availability

All data used to support the findings of this study are included within the article.
